# Manual Complet De Médecine Légales

**Published:** 1859-11

**Authors:** 


### NORTH AMERICAN

### MEDICO-CHIRURGICAL REVIEW.

**NOVEMBER, 1859.**

### Analytical and Critical Reviews.

ART. I.—*Manuel Complet de Médecine Légale, ou Résumé des Meilleurs Ouvrages Publiés jusqu'a ce Jour sur cette Matiere, et des Jugements et Arrets les plus Recents.** Par J. BRIAND, D.M., de Paris, ex-Professeur d'Anatomie de Médecine et de Chirurgie; et ERNEST CHAUDÉ, Docteur en droit, Avocat de la Cour d'Appel de Paris; Contenant un Traité Elementaire de Chimie Légale, dans lequel est discuté la Marche à suivre dans les Recherches Toxicologiques et dans les Applications de la Chimie aux Diverses Questions Criminelles Civiles, Commerciales et Adminstratives. Par H. GAULTIER DE CLAUBRY, Docteur en Science, Professeur à l'École Superieur de Pharmacie, Officier de la Legion d'Honneur, etc. etc. Sixième edition. Avec 3 Planches gravées et 64 Figures dans le Texte. Paris: J. B. Baillière et Fils, 1858; pp. 948, 8vo.

*A Complete Manual of Legal Medicine; or, a Digest of the Best Works Published up to the Present Time on the Subject, and the most Recent Decisions and Decrees.* By J. BRIAND, M.D., etc., and ERNEST CHAUDÉ, Counselor-at-law and Advocate in the Court of Ap-

* There follows on the title-page: "Precedé de considerations sur la recherche et les poursuites des crimes et des delits, sur les autorités qui ont droit de requérir l'assistance des médecins ou chirurgiens, sur la distinction etablie par lui entre les docteurs et les officiers de santé, sur la maniere de proceder aux expertises medico-legales, sur la relation des rapports et des consultations, sur les cas ou les hommes de l'art sont responsibles des faits de leur pratique, et sur les honoraires qui leur sont dus, soit en justice, soit dans la pratique civile; et suivi de modeles de rapports et de commentaires sur les lois, decrets et ordonnances qui regissent la médecine, la pharmacie, la vente des remèdes secrets," etc.

peals, Paris: Including an Elementary System of Legal Chemistry, etc. etc. By GAULTIER DE CLAUBRY, Doctor in Science, and Member of the Legion of Honor, etc. Paris: Baillière & Son, 1858.

THIS "Manual" is the latest, and may in some respects be regarded as the best among the systematic treatises which have appeared in France during the last half century, on the science, known in the United States and England under the title of Medical Jurisprudence, and on the continent of Europe under that of Legal Medicine, and, occasionally, as Forensic, State, or Juridical Medicine. The sight of a portly tome of nearly a thousand pages octavo, composed and ushered into public notice by its well-known author in chief, and his two aids in law and chemistry, may well bespeak our respect, and create a desire for intimacy with its contents, ample and diversified as they are. We must, at the same time, confess that we cannot refrain from looking with a feeling akin to veneration at a ponderous folio of upwards of twelve hundred pages, black letter, which is no less a work than that of the celebrated *Questiones Medico-Legales* of Paulus Zacchias, and which, in its antique parchment binding, is now lying on our table, alongside of the somewhat undressed modern one of M. Briand, with its rough sides and paper cover. Our design in thus introducing with so little ceremony the Roman work of 1621 and the Parisian of 1858 to our readers is, that we may with the better grace tell them of some passages in the history of medical jurisprudence, the scientific or clearly speaking age of which is very nearly included between these two dates. The continual neglect of the subject in most of our medical and law schools, so far at least as a failure to teach it with any approach to method or fullness, renders it imperative on reviewers and journalists to lose no opportunity to give their aid in supplying the omission.

Medical Jurisprudence has been variously defined. We would call it, that science which teaches the application of every branch of medicine to the elucidation of questions of law and right, which come before the regular tribunals of the country for adjudication. We see no good reason for including under the head of Medical Jurisprudence the subjects of Public Hygiene and of Medical Police, on the ground that the two last are regulated by laws, in the framing of which a knowledge of medical science is of paramount importance. The enactments for their observance are precise, as is the penalty for a breach of them; and although in some cases, as nuisances, for example, they seem to make a near approach to the domain of medical jurisprudence, yet the objects aimed at and the results reached are, in the main, widely different, the one from the other. In giving this opinion, we are in opposition to such authorities as Foderé, and Paris and Fonblanque, the two latter of whom representing each respectively, medicine and law, united their lore and experience in composing a work on medical jurisprudence, in which they treat, also, of the public health and medical police; but we have at the same time, the majority of medico-juridical writers on our side.

Medical jurisprudence is a science of modern origin, for we can hardly speak of its separate existence before the beginning of the fourteenth century, when anatomy was first studied on the human subject, nor of its acquiring distinct outlines before the sixteenth century, by the publication, in 1532, of the *Carolinian Criminal Constitution** of the Emperor Charles V. Not only did articles 147 and 148 formally admit the indispensable necessity of medicine in aid of medical jurisprudence, but many other articles also, in apportioning to a certain extent the punishments to the physical effects of crimes, imply of course a rigorous appreciation of these effects. They who are disposed to enter into the literature of the subject, may consult the book of Deuteronomy, chapter xxii. verse 15, and the *Lex Aquilia* of the Romans, and enactments on certain points—such as legitimacy, abortion, the relative fatality of wounds, etc., extending from the reign of Numa Pompilius to the times of the emperors Severus, Antoninus, Adrian, and Aurelius; the edicts of these latter being based on the writings of Hippocrates, Aristotle, and Galen. By the Aquilian law, it was decreed, that if a slave have been wounded, but without the wound being necessarily mortal, and that death takes place through negligent treatment, an action can only lie for the wound and not for the death. The case here supposed, is that of a simple wound without premeditation, and not one coming under the charge of assassination. Of the ancient writers, Galen is the only one whose works furnish some materials for legal medicine. We may mention more particularly his treatise on "Feigned Diseases," and his remarks on the lungs of the fœtus and of the adult, remarks which must be regarded as the first data toward pulmonary docimasy, or the hydrostatic test. A nearer approach to system than could be obtained from these scattered fragments, seemed to be made by the Justinian code, in which are found regulations respecting marriage, the duration of pregnancy, supposititious children, impotence, etc.

The influence of Roman law was not lost on the rulers who governed in Western Europe after the downfall of the empire; and accordingly

---

* "*Constitutio Criminalis Carolina*, which still forms the basis of the criminal proceedings of the German law courts. In the Code, it is enacted that medical men shall be consulted whenever death has been occasioned by violent means, whether criminal or accidental, by wounds, poison, hanging, drowning, or the like, as well as in cases of concealed pregnancy, procured abortion, or child-murder," etc.—PARIS AND FONBLANQUE, *Medical Jurisprudence*, vol. i. p. 10: Introduction.

we find Charlemagne, in his *Capitularies*, requiring, under specified circumstances, the intervention of medical men, and directing in a forcible manner, as Justinian had previously done, the appearance of regular medical experts before the tribunals. In the long night of intellectual darkness which followed the death of this sovereign, medical jurisprudence, in common with all the sciences and literature, was lost sight of; and its appearance with distinctive attributes must be referred to the period already indicated, which was that of the real study of anatomy. The first methodical work on our science is due to the eminent French military surgeon, Ambrose Paré, whose presence in the army was deemed equivalent to a reinforcement of many thousand men. His work, dated 1575, is on "Reports:" in it the author speaks of feigned diseases. In the last part of this century, and in the beginning of the next, medical jurisprudence made decided progress, both in France and Germany. It was indeed time that medical science should enlighten legislation, and interpose between the cruelty of the law and its innocent victims. Pigray, surgeon to Henry III. of France, and a contemporary of Ambrose Paré, relates a case brought before the parliament of Paris, which, at the time (1589) was sitting in Tours. Pigray, in association with three physicians of the king, went to visit fourteen persons, men and women, who had made an appeal from a decree of death for the crime of sorcery. The reporters state that they made the desired examination into the bodily and mental condition of these unfortunate creatures; some of whom were averse to dying, and others desired it. The opinion of the medical jurists in this case, was to ply them with hellebore in order to purge them rather than subject them to any process of a penal character. It is an evidence of the limited view which then prevailed of the attributes of medical jurisprudence, that both by Henry IV. and Louis XIV., a surgeon alone was appointed in each town and populous district, to make a report on the killed, wounded, or otherwise mutilated. The appointment rested with the chief physician of the king, and was often made to favorites or venal applicants, without regard to qualifications. In 1611 appeared the Discourse of Vincent Tagereau, on Impotence in Man and in Woman, in which the author insists on the indecency as well as the insufficiency of the congress, (*congressus,*) or the test of coition between husband and wife, in the presence of a designated number of surgeons and matrons; for such was the law when an application was made, on either side, to dissolve the marriage tie, on the plea of impotence. This odious exhibition, alike adverse to morality and physiology, was not abolished by law until the year 1667. One of the earliest works on a subject of medical jurisprudence in Germany, was published at the very close of the sixteenth century, (1599.) Its title, *De Cruentatione cadaverum injusta cæde*

*factorum præsente qui occidisse creditur*, sufficiently indicates its real value; the author, Andrew Libavius, upholding the absurd belief that the wounds of the dead man will bleed when the murderer is present. If it were true that the state of a science in any country should be measured by the literary labors to which it has given rise, Sicily must be regarded as the mother-land of medical jurisprudence, since it was at Palermo, in 1602, that Fortunatus Fidelis—what a name of happy omen!—collected and published the first treatise on nearly all that was then known of the medico-legal doctrines of his time. His work, *De Relationibus Medicorum*, consists of four books, the first of which is on subjects of hygiene. We notice, with a passing remark on the waste of mind and learning displayed in it, the voluminous production of Buonaventura, entitled *De Natura partus octomestris, adversus vulgarem opinionem.* This is a folio volume containing upwards of one thousand pages, devoted to the topic of eight months pregnancy, with an account of all the discussions that have arisen on the legitimate period of utero-gestation.

About twenty years after the publication of the treatise of Fidelis appeared the more celebrated and still esteemed Medico-Legal Questions* of Paolo Zacchia, or, as he is more usually called by his Latinized name, Paulus Zacchias, a Roman of a noble family, who was versed in literature, and withal a poet, a painter, and a musician. To Zacchias must be awarded the credit of being the first to exhibit medical jurisprudence as a science; and we should hardly find fault with the teacher whose retrospective lore did not not carry him beyond the date of publication of the *Questiones Medico-Legales.* A copy of the Frankfort edition, 1688, now before us, bears on a fly-leaf the title of *Questionum Medico-Legalium Opus Absolutissimum in Tres Tomos Divisum.* It is a ponderous folio, bound in parchment, and consists of upwards of twelve hundred pages, in three volumes. The work, apart from its intrinsic merits, is a literary curiosity by the prefixes, interpolations, and other additions of its editor, *Georgius Francus*, whose lavish praises of the author are exceeded by those of which he is himself the subject, on the part of admiring friends and correspondents. Some of our medical critics would be disposed to temper the severity of their censures of American physicians who edit English works, were they to look over the title-page, preface, and other preliminary articles on the Medico-Legal Questions of Zacchias, introduced by their Heidelberg brother. We subjoin in a

---

* Questiones Medico-Legales, in quibus omnes materiæ medicæ quas ad legales facultates videntur pertenere, proponuntur, pertractantur, resolvuntur. Libri ix. The first of these books, or, as they would perhaps be now called, fasciculi, was published in Rome in 1621. The eighth and the ninth book appeared in 1650.

foot-note the contents of the title-page,* as found in the original. The artistical effect on the eyes of our readers must of course be lost, from our inability to give the variety of type and alternate black and red letters which embellish the page. Notwithstanding so stately a presentation and so formal a dress, the literary magnificos did not deem it beneath them to speak of the work being so very necessary (*plus maxime necessarium*) to the skilled in law and medicine, as well as to those holding seats in the ecclesiastical courts, in terms which, now-a-days, the publisher is content to append in the shape of an advertisement. After the title follows a dedication to the Prince Elector Palatine, and on a third leaf is an address setting forth the merits and deeds of the Prince and his family. Next we come to the preface, written also by Georgius Francus, who, after the example of James Dumascene, compares medicine to the ocean; hygeia reminding us, why one cannot say, of the Atlantic; therapeutics of the Indian Ocean; and so on. And, again, the art of medicine is compared to a ship, of which reason is the prow—one would have said rather the rudder—experience the poop, surgery the oars. By another of these *concetti*, the four cardinal points were made to represent the four sects in medicine, viz.: the dogmatics, the empirics, the methodists, and the hermetics, or alchemists.†

Editions of Zacchias had appeared first in Rome, and then in Leipsic, Amsterdam, Leyden, and finally Frankfort, the last edited by Horstius. Bencard, the publisher, wishing to bring out a new edition, to be enriched with commentaries by jurists, as well as by physicians, applied to Frank,

---

* Pauli Zacchiæ Romani, Totius Status Ecclesiastici Protomedici Generalis, Questionum Medico-Legalium, Tomi Tres. Olim aucti et emendati à viro celeberrimo Joh. Daniel Horstio, nunc pluribus in locis notis et Observationibus curiosis qua Juridicis, qua Medicis, quas benevole communicarunt, celeberrimi Icti et Medici, Quorum Catalogus post Prefationem videre est, illustrati, emendati atque adaucti a Georgio Franco, Med. et Phil. Doct., P. P. Universit. Heidelberg. Pro-Cancellario, Facult. Medic. Seniore, Diverss. S. R. I. Electorum ac Principium Archiatro, C. P. Cæsar, Academ, Natur Curioss. Argo a et Italicæ recuperatorum Collega. Opus omnibus Juris, utriusque Medicinæ peritis, nec non Tribunalium, (Ecclesiastici Civilis,) Assessoribus, maxime necessarium. Cum gratiâ et privilegio Sacræ Cæsar. Majestatis. Francofurti-ad-Mænum: Sumtibus Johannis Melchioris Bencard, MDCLXXXVIII.

† Reference is made, in encomiastic terms, to the work of Fortunatus Fidelis, and a double literary fraud, of which it was made the subject, is revealed by Francus. It seems that an anonymous writer, a crafty old fox, (*veterator*,) put forth the work of Fidelis, under the title of *Schola Jureconsultorum Medica, Relationum, Libris Aliquot Comprehensa*, as the production of Reinesius of Altenburg, in Saxony, a well-known professor and man of letters; and, in order to give the affair a greater show of truth, a preface was introduced, as having been penned by Reinesius. The fraud was exposed by Merklin, who showed, among other things, the great difference in the style of the imputed and of the real editor.

who forthwith set about enlisting the aid of men eminent in law and medicine. Their names are recorded and their merits duly emblazoned. We shall not repeat the enumeration nor the specifications of the different chapters of the work, to which they added notes and commentaries. They are all mentioned in the preface, and constitute a list of (*præclarissimi, celeberrimi, honoratissimi, præstantissimi*) men who have left behind them no enduring marks, unless we except the matter which adheres to the original work of Zacchias himself. Every age furnishes a similar lesson of rebuke to vanity and ambitious aspirations. While we receive its teachings, we must not, however, be deterred from continually exerting ourselves in the path of improvement. The exercise is a salutary one, even though it may not lead to the temple of fame. But not alone were the volunteer literati of the day enlisted by Francus, the most erudite of editors—he presses into the service of legal medicine all the philosophers, all the poets, historians, satirists, essayists, pedagogues, and dreamers, numbering more than a thousand, a catalogue of the names of whom is given. After the preface, we read a life of the incomparable archiater, or chief physician of the Pope, Paulus Zacchias, by the most noble, *magnifico*, most excellent and skillful Georgius Francus, who, by others of his admirers, is called, in Latin verses commemorative of his fame, a Hippocrates, a Podilarius, a Galen:—

Hic vir, hic ille scrutator Justiniani

Mire conjungens *præcipe* cum *recipe*.

No offence can be taken at the length of the life of Zacchias, which occupies but three pages; one of these is filled by an enumeration of the successive appearance of the nine books of legal medicine, and of other works from his pen. The biography opens with a quotation from Aristotle on the hereditary transmission of mental qualities, to the effect that the good spring from the good; and of this Francus thinks that a proof is furnished in the fact of Zacchias springing from a noble family in Rome. Boethius is cited to show that good blood makes virtue to shine more conspicuously. A passage from Ovid's Metamorphoses is next introduced, expressive of the wonderful powers and successful labors of the Roman medical jurist, whose fame is represented to be allied with that of Rome herself. After all this heralding, the actual narrative of the life of Zacchias is summed up in a few lines, communicated to the editor by his friend Albertus Guntherus Saxo, and amounts to little more than the fact, that the author of the celebrated *Questiones Medico-Legales* was born in Rome, A.D. 1584, and died in 1659, aged seventy-five years; having filled the offices of first physician to the Hospital of San Spirito, to which he was appointed by Pope Innocent X., and of physician to the pontifical palace by Alexander VII. Of the ways and manners of the man, the superlative Francus says nothing, which, considering the various tastes and accomplishments of his professed idol, must be a matter of regret to all who like to see or read of genius in its hours of ease and in dishabille. All that the German pedant allows us to imagine of Zacchias out of his study, is his grave walk and oracular prescriptions, in his rounds through an immense hospital, and his obsequious homage to two popes.* We should have been unable to make even incidental mention of the varied accomplishments of the great Roman physician, but for the information in the page given to him by Tiraboschi.†

In the midst of the eulogistic offerings to the merits of Zacchias, we meet with two short addresses from himself; one to the medical reader, the other to the learned in the law, invoking their kindly reception of his work and telling of the pains taken in its preparation. To the former he holds out the language of advice, and recommends him to pay more attention to what is spoken, than to the person who speaks. As evincing the sincerity of his intentions in preparing his work, he takes God and man to witness, that he was intent only on collecting what was true. The legal reader is addressed in terms of propitiation, as if the author had ventured on ground with which he was not familiar, and on which his points of law might be commonplace, if they were not erroneous.

The work of Zacchias, divested of its wrappings and bedizenings, is still somewhat of a parti-colored character, and embraces a number of themes not related to the main subject. It evinces a spirit of scholastic subtlety which was so prevalent in that age, and which, although luxuriating most in the field of theology, displayed itself in every department of literature and even of science. Thus, for example, under the head of *Questio de Amantibus,* we have a dissertation on Love and Melancholy, and an inquiry into the seat of love, and whether it is in the brain, the heart, or the liver. Miracles being the theme, the Roman author asks:

---

* The other productions of Zacchias are—I. On Quiet in the cure of diseases. II. Sudden and unlooked-for deaths, and their precognition, and the precautions required. III. Hypochondriacal affections, written first in Italian and afterwards translated into Latin by Khone. IV. On the Oriental opobalsam. V. On the Miracles recorded in Holy Writ, in their physical relations. This last is mentioned by Bardi in his eulogy of Zacchias, who, as we learn on the same authority, delivered discourses on the following subjects, in his native tongue: On the passions of man, and the evils to which they give rise, and of their cure by physical as well as moral means; on beer and fermented liquors; on contagion, in which it is shown that there is much less danger from this cause than is generally imagined; on Lent, and the means by which it may be kept without danger to health; on kissing; on laughing and crying.

† Storia delta Letteratura Italiana, tomo viii. p. 324.

What is a miracle? He next treats of prophecy, of miraculous cures of diseases, and miraculous deaths; giving examples of each. Still on theological ground, he speaks of fasting and Lent. In another section, in imitation of Hippocrates, he discourses on "Air, Waters, and Places." In a fresh chapter, he mentions the cloaques and the aqueducts of Rome. On the subject of matrimony, he insists on larger marital privileges than would be at all consistent with the charter of the Woman's Rights school of the present day. Thus we are told, that a woman who refuses to go to the dwelling of her husband, commits a mortal sin; that a man may castigate his wife mildly and in moderation, especially if for cause; and that a woman cannot be said to be unreasonably punished by her husband if he inflict on her a cuffing and slaps, and do not beat her with a stick or a cudgel. It is also conceded to be part of the husband's privilege to persuade his wife, by a marital discourse, to make him her heir. The husband's assertion of his having had carnal connection with his wife must prevail over her denial to the contrary.

The first of the three volumes of the work consists of five books, each of which latter is divided into what we shall call sections, (*Tituli*); then again into "questions" or subheads of the several subjects which come up for discussion. The four sections of the first book treat of Age, in its several periods, from infancy to senility and decrepitude; Parturition, legitimate and vital; Pregnancy, Superfœtation, and Moles; Cause of Death from Delivery, and Cause of this last; Resemblances and Dissimilitudes of Children to their Parents. The second book, in its four sections treats of Dementia, and Lesion of the Reasoning Faculties, and all the Diseases which disturb Reason; Poisons, Sorcery, and other Congenerate Matters; Various Questions touching Bodily and Mental Infirmities of a Slave or Servant, which would render null and void the sale or hire of such Slave or Servant. Book third has three sections, which relate severally to Impotence, Feigned Diseases, Plague and Contagion. Book fourth treats of Miracles, Virginity, and Rape*—an odd conjunction of subjects, and which might furnish matter for satirical remarks. In the fifth book we read in four sections, about Fasting and Lent; Wounds; Mutilation of the different members of the body, of which the virile one is declared to be the chief, (*pater est omnium membrorum;*) Air, Waters, and Places. Zacchias, it will have been seen, and also, as we shall show hereafter, does not make any attempt at a natural division, or the relation of one to the other, of the several subjects of which he treats. The last

* Question fifth of this section is given to the subject of bestial violence done to the youth of the male sex. "Stuprum non modo in puellas et virgines et viduas committitur. Alciat et alii apud Carrar *de med, part* ii., No. 200, sed, à scelesitissimis hominum, Naturâ abutentibus, etiam in masculos patratur."

question of this fifth section is on Sewers, Aqueducts, Canals, Gardens, Orchards, Furnaces, Dung-heaps, Privies, Stables, and offensive Manufactures. All of these topics are discussed in relation to their influence on the public health, and hence they come under the head of hygiene rather than of medical jurisprudence.

The second volume begins with the sixth book of the entire work. The first section is on the Wrong-doings of Physicians which are punishable by law. Its contents may hereafter engage our notice in connection with medical ethics, to which rather than to medical jurisprudence it more especially belongs. Still we must not pass it over in silence on the present occasion. Zacchias wrote on this theme in conformity with the expressed wishes of some of his friends. It is not his intention, he says, to speak of all the shortcomings of medical men, but only of those which are committed through malicious intent, or from negligence and ignorance, in the treatment of the sick, and which subject the offending party to the penalties, not only of the civil, but also of the canon law. The author enters his early protest, however, against confounding physicians who practice on rational principles and according to the teachings of philosophy, with the herd of pretenders who dabble in drugs, and busy themselves in prescribing for the sick or in practicing minor surgery, especially phlebotomy, and with another set who deal in aromatics and balsams, and also empirics and chemists, (alchemists ?)* and the whole brood of quacks and mountebanks. Not alone does the male sex furnish the entire list of irregular practitioners. Silly women also play their part, either in administering to the sick or in dispensing medicines, or in exercising the obstetric art. Were Zacchias living at the present time, there is reason to believe, from these remarks of his, that female colleges of medicine would find little favor in his eyes. One of the commentators of the work enlisted by Francus, quotes Pliny's complaint, that, in this art alone, any man setting himself up for a physician is immediately credited, although the danger is greater from deception in this than in other cases. The law had no punishment for gross ignorance; there was no penalty to warn of the danger occurred; and the doctor was allowed to kill men with impunity. At the present time, and in our own happy land, the law interposes no obstacle, inflicts no punishment on any knave who volunteers to cure disease in total ignorance of its nature and of the functions which it deranges, and whose impunity is the greatest when his pretensions are the most extravagant and absurd. It is only the regular and educated physician or surgeon who is mulcted in damages to feed the cupidity of a patient whose obstinate dis-

* Chemistry proper, as a science, scarcely had existence at this time.

obedience to professional directions were chiefly instrumental in bringing about the failure of the remedial means employed.

The circumstances in which malicious intention to injure or destroy a sick person confided to his charge, or in which the physician acts in a negligent manner or through ignorance, are examined in detail. The very thought of a medical man being so utterly faithless, base, and cruel, as not only to prolong the period of the disease of a person who trusts his life in his hands, but also to administer drugs which will produce an aggravation of the suffering of the patient, and death itself, is so abhorrent to every feeling of humanity, that, in this age of the world at least, or at any rate among civilized and Christian people, its occurrence can hardly be thought possible. This is one of the monstrous crimes on which the laws are silent, for the reason that their perpetration is deemed to be without ordinary motive, object, or excuse; in fine, so monstrous as not to be put on ordinary record, for fear of contamination and evil suggestion by the very naming of them. If the Roman physicians of the regular class were open to such imputations in the time of Zacchias, we have reason to congratulate ourselves on a purer morality at the present day. No code of ethics, however minute it may be in telling what the physician is to do and what not to do, in his intercourse with his patients, hints even at such a gross infraction of divine, to say nothing of human law. We cannot, we fear, speak with equal confidence of the freedom of all the rank and file of our profession in these modern days from the two other charges to which physicians are said, in the work before us, to be sometimes amenable. These are, as previously stated, ignorance and negligence. Six specifications of the display of ignorance are adduced; some of them the result of self-sufficiency and rashness; others are manifested in wrong pathology and therapeutics, but the error in some of these is made to rest on theoretical grounds. To direct mild remedies when an active treatment is required, and to urge the latter when the former would be sufficient, are specified among the errors from ignorance. Here and there we glean a knowledge of the favorite treatment of a disease, which is not without interest; as, for instance, when certain physicians are charged with dereliction of duty in neglecting to draw blood in dysentery with inflammation; the remedy being clearly indicated by the pain, the fever, and the inflammation, but withheld by them on account of the intestinal flux. Ignorance is also charged on the physician who attaches undue importance to a prominent symptom, while neglecting the essential characters of the disease, and, on the other hand, omitting to treat a troublesome symptom by any special remedy, under a belief that it will follow the course of the main disease. Zacchias tells, on this occasion, of the case of a man who, while laboring under a simple tertian fever, was, at the same time, much distressed with cardialgia. His physician rejected the advice of the author to give the patient food just before the accession of the paroxysm, with a view of protecting the cardiac orifice from the irritation of acrid bile; and the consequence of this obstinacy was a fatal termination of the disease. The observations and teaching of Torti, at the beginning of the next or eighteenth century, shed so much new light on the nature of the complications of organic disorder with periodical fever, and on the necessity of large doses of bark for treating these cases with success, as to convince every modern practitioner that neither Zacchias nor the younger and self-willed physician with whom he was brought in contact, was correct in his pathology and in the indications of cure. The case was one of those malignant or pernicious intermittents, our American congestive fever, marked by cardialgia, and which called for a more vigorous treatment than would seem to have been indicated by the apparent simplicity of the fever, or the gastric disorder which was combined with it. We must not, however, omit to repeat the assertion of the Roman author, that he had seen persons with these fatal symptoms snatched, as it were, from death, by administering food on the accession of the paroxysm. Galen is cited as corroborative authority.

Addressing himself more particularly to an exposure of the errors of regular physicians, especially in as far as they relate to morals and science, Zacchias adverts to the harsh strictures on medical men, in which some writers have indulged, but which are more particularly applicable to greedy pretenders. Avarice, so deeply implanted in the human breast, must, of course, find its place among physicians in common with the rest of mankind. The examples of this vice in the profession are, however, few, and the very reverse quality, or disregard of personal interests, is the prevalent sentiment. Envy and detraction among medical men are accusations not so easily disposed of. Associated with ignorance are the confident promises by a physician of the speedy restoration of his patient to health. The empirical tribe is particularly lavish of these promises, safely speculating on the ignorance and fluxibility, to borrow an expressive word from the original before us. Garrulity and gossip, under the head of loquacity, are cited as faults of medical men, when they extend to a revelation of the infirmities and secret personal and family details of their patients. There are, however, limits to professional reserve in such matters laid down by law, of which we shall speak at another time.

Very decided language is uttered on the subject of one medical man consulting with another respecting the treatment of a patient. Thus we read that a physician, who, in a case of doubtful issue, refuses to consult with another member of the profession, commits a mortal sin, (*mortaliter peccat.*) The preference of a friend, or one whom he knows, over another, who is a distinguished practitioner, by the physician in attendance, is referred to as a matter of doubtful propriety. But, on the authority of Silvaticus, a physician commits a mortal sin, who, from hatred or envy, refuses to meet in consultation a more learned associate, or who, through friendship or any other feeling, calls in a person of inferior qualifications. The force of this denunciation is lessened not a little by what follows, viz., that by canonical law not only is the physician not blamable for refusing to consult with a Jew, or with one who does not believe in the Christian religion, or even a heretic, (not Catholic,) but his doing so would be a mortal sin. Under the head of errors of omission, relating to the advice to be offered by a physician, a number of questions are brought up for adjudication; such as recommending the sick to confess and receive the rites of the church, prescribing for the sick who are living at a distance, the utility of coition in the cure or prevention of any disease, the effects of virginity on the bodily health, the consequences of ebriety, the extent of the claims of the public and of the sick on medical men. Errors of commission in the practice of medicine are also passed in review. Next are treated of, in some detail, the errors of commission and those of omission on the subject of fees, and the claims on the professional services of a physician by those who are unable to remunerate him. Then follow discussions on various questions relating to the conduct and misconduct of surgeons and the like craft, and of dispensers of aromatics and of salves and unguents, and of obstetric grannies, and, finally, of the assistants and nurses in the sick-room.

We pass over the second section of the sixth book, which treats of Tortures and Punishments. Among other evidences of our living in a more humane age than our forefathers is the abolition of torture, and hence we are only called on to speak of it as a purely historical question. In this light it does not demand, at the present time, any notice from our hands. Nor can we dwell on the matter of the next section, which consists of an inquiry into the right of precedence between the physician and the jurisconsult or lawyer, as he is now-a-days more generally termed. The subject is not without some amusement for those who take either side of the question; they will find sketches of professional men drawn in other than very flattering features. It is true that the objections arrayed against medicine and doctors are met by the author in due form, and the same must be said of the practitioners and professors of law. Men will argue from the last rebutting testimony, but there is ill nature enough in all of us to secure a lively recollection of the accusations and slander on the other side. The actual and relative merits of medicine and law are the subject of the concluding subsections, or "Questions," as they are called in the volume.

The seventh book opens with a section on spinsters, including hermaphrodites, which is followed by one on the Divine offices, or rather on the infirmities and diseases which excuse or prohibit their exercise. Curious questions make up the third section of this book. They run upon conjugal debt or obligations, relating solely, however, to sexual congress; and are examined and argued with a gravity and details quite puzzling to all our present received notions of delicacy, if not of ethical propriety. We care not, therefore, to follow the author in his replies to "*Quando, quantum, quomodo debitum conjugale sit reddendum?*" nor to the other respecting the circumstances which excuse husband and wife from the reciprocal payment of these important dues. A dissertation on female semen in this section gives occasion, or rather opportunity, for a great parade of useless learning, both medical and theological, including the subject of the "immaculate conception." Sanchez and other canonists indulge in minuteness of detail on the union of the sexes in married life, which are not called for by any consideration of duty or of piety, but which are, on the contrary, as repugnant to physiology as they are to common delicacy. Zacchias is very free in his quotations from this class of writers, to imitate whom would now properly subject the offender to punishment for erotic details, *contra bonos mores.*

In the fourth section of the seventh book the author treats of Marks, (*Stigmata,*) including under this head all those spots, stains, and scars seen on the skin of different persons; some of which are of intra-uterine origin, others the result of wounds and diseases, and occasionally they are caused by artificial processes.

The initial section of the eighth book contains no less than twenty-four subsections or questions on "Irregularity," which, as understood by the canonists, is that deviation, either of the body or mind, from its normal standard, which unfits a person for discharging the sacred offices of the church, either on account of congenital defects or subsequent disease, mutilation, disfigurement, or personal impurities. Under the head of diseases of the spirit the canonists rank epilepsy and apoplexy. In speaking of spiritual irregularity the question comes up: whether the practice of medicine contributes to this state? The reply of the author is in the negative, although the temptations to err are great. The hereditary tendency to epilepsy is alleged to be greater on the side of the mother than that of the father. A detailed account is given, under separate heads, of the disorders and malformations of the different organs. Gout and arthritis are also spoken of in this connection, and then itch, leprosy, syphilis, (*morbus gallicus*, as it was called,) fever and the plague.

The subject of the second section of this book is on Medical Remedies, and contains four subsections or questions, viz.: I. What are medical remedies, what persons are liable to err in the use of them, and is the patient bound, and in what cases, to obey his physician? II. Dietetics. III. Pharmaceutics. IV. Surgery. Medicine works by three sets of agents, viz., diet, pharmacy, and surgery. In the use of remedies blame is attributable, at one time, to the physician, at another, to the sick person, and again, to the attendant or nurse. The first may fail to foresee what ought to have been foreseen, or he may imagine that which does not come to pass, or grant what ought not to be granted, for the sake of gratifying the patient. More commonly this latter personage is to blame in not only not obeying, but rather in spurning the advice and prescriptions of his physician, in order to satisfy his own desires, or from dislike to bear the discomforts of sickness. The attendant sins in not carrying out the directions of the physician, or in giving improper food and drink to the sick person, and sometimes in advising a recourse to means, the very opposite of those which had been regularly prescribed. The extent to which the patient may be supposed to be bound to conform to the advice and directions of his physician is discussed with earnest particularity. Let a sick person know then (*Zacchiâ loquente*) that he who rejects his doctor's advice and medicines, not only acts in contradiction to a proper regard for his own body and to the sacred page, but also against the intentions of the Omnipotent Deity who created medicable things out of the earth for man's health and well-being. Man, as the custodian of his own health, is bound to avail himself of all the means offered by Nature for the purpose of preserving it. The patient is not excused for disobeying his physician on the ground of the disagreeable properties and harsh operation, or expensiveness of a remedy. Original antipathies and idiosyncrasies of the patient must, however, be respected by the physician.

In the third section of the eighth book, under five subheads or "Questions," the author inquires into the effects of convent life on nuns, and the diseases which would justify or require their abandoning it. There were only three causes allowed by papal rescript for a nun leaving her convent, viz., for fire threatening destruction of the building and the life of the inmates, epidemic pestilence, and leprosy. It is only with the two last that the physician has to deal, and these, accordingly, are made the subject of disquisition by Zacchias, in his usual painstaking minuteness of detail and love of scholastic refinement. Something we glean, on the occasion, respecting the visitations of epidemic disease and the states of the air at the time of their prevalence, and also in regard to plague and contagion, and the extent of range of this latter. Frequent reference is made to the regulations laid down by the authority of the Popes in such matters, so far as they are supposed to affect these recluses, and to furnish reasons for their temporary withdrawal from the convent, not alone for the sake of the individual, but, also, for the protection of the other inmates. The author inquires into the probable necessity of other diseases, besides those specified above, furnishing cause for leaving the convent. He expresses his surprise that writers like Sebastian de Soto, should regard syphilis, ulcerated cancer, scrofula, and ophthalmia as sufficient to authorize removal, while they overlook entirely such a terrible disease as epilepsy, and the danger of the spectators and by-standers being led to sympathetic imitation of the one first taken with the convulsions. The mention, by an orthodox Catholic and a Pope's physician, of syphilis, among the diseases of females who had made a vow of perpetual chastity, was calculated to give additional strength to those reformers who denounced the impurities and vices which had become, unhappily, too common in monastic life. Long before Zacchias wrote, Boccaccio had exposed them in his Decameron.

The ninth and last book is given to the following subjects: the time when the fœtus becomes an animated being; exsection of the fœtus, or the Cæsarean section; the impediments to copulation and generation; the resigning of benefices; the period of segregation of those suspected of having the plague, in the inquiry into which we find a discussion on plague and contagion, as an appendix to the third section of the third book; the question whether the making of silk during the prevalence of the plague should be prohibited as an offensivve manufacture, (*ars immunda;*) on tobacco, on chocolate and brandy, (*aqua vitæ;*) those diseases which prevent the receiving of the Eucharist; dissolution of matrimony, divorce, separation from the connubial bed, and freedom from espousals; the female semen; in the order of nature, is the first born the first conceived? and in the case of twins, which is to be supposed the one born first? The reader will wonder at the variety of the themes and their odd and unexpected collocation, as evinced in these contents of a single which is the concluding book of the second volume. They are introduced and discussed with good faith, and in all sincerity and gravity. The same may be said of the entire work; and on this account as well as on that of the importance of many parts of it, and from its being inaccessible to the great body of our readers and to the profession at large, we have been induced to give, probably, the first analytical notice of the *Questiones Medico-Legales* in the English language; nor have we seen the like in any other to guide us in our task. But we are anticipating what might be more appropriately said after our notice of the contents of the third volume. These consist of eighty-five answers or opinions, by Zacchias, on medico-legal questions: they partake of the general character of the contents of the body of the work—profuse learning and copiousness of detail; but, it must be added, they evince also great perspicacity and clearness of argumentation. Some of the *Consilia* of the author are quoted in modern works, and all of them possess a certain degree of value as curious medical, if not always medico-legal histories of rare cases. That of Casali affords a good specimen of the manner in which Zacchias handles his subject, in his bringing to bear a large amount of erudition on the causes which affect and alter the physiognomy, and make the question of the recognition of personal identity very difficult and sometimes impracticable. Foderé, one of the best writers on legal medicine, has followed closely the line of argument and illustration traced by Zacchias, in the case of Casali, respecting the changes in the human countenance and form, caused by climate, diet, hardships, mental emotion, and age Did space permit, we would cheerfully place on these pages a translation of some of the "opinions" of the author. We hope, at any rate, to be able to refer to them again before we have done. In addition to the "opinions" of Zacchias himself, there are decisions, one hundred in number, on the same and similar topics, by the *Rota Romana*, or Court of Appeals,* which conclude the work.

Were we so inclined, it would be easy for us to indulge in a parade of bibliographical lore, by introducing a long list of writers on medical jurisprudence, from the time of Zacchias to the present day. It will be sufficient, however, for our immediate purposes, to refer to the two volumes of Wildberg, now before us, entitled *Bibliotheca Medicinæ Publicæ*, the first of which contains a list of all the works, in the shape of systematic treatises, essays, and disquisitions on particular branches of forensic medicine, covering a period of nearly three centuries, or from 1533† to 1818,‡ and classified according to subjects. They number little short of 3000, (2980.) The second volume contains a correspondingly copious catalogue of all the writings on political medicine or public hygiene, amounting to upwards of 2000 in about the same period. Orfila, in the beginning of his work *Leçons de Mêdecine Legale*, gives the titles of one hundred and thirty-three works on the science, brought out during the first third of the present century. In the brief period which has elapsed from that to the present time, systematic works of reputation, and a large number of papers on different branches of the science, have been published. To some of these we shall have occasion to refer as we advance in the present article.

But while we decline to test the patience of our readers by a long enumeration of names and titles, it is but due to the subject and to them that we should continue our historical outlines of medical jurisprudence. Towards the middle of the seventeenth century, and not many years after

* The "Rota Romana" has a collegiate constitution, and consists of twelve persons, of whom three must be Romans, one a German, one a Frenchman, and one a Spaniard.

† L. Guarici—De conceptu natorum et partu septimestri.

‡ A. Hencke—Abhandlungen aus dem Gebiete der gericht lichen Medizin.

the first appearance of the great work of Zacchias, France could point to two productions on the subject—that of René Gondri, (1650,) and that of Nicholas Blegny, (1684,) which was followed by the more useful work of Devaux, (1701.) These, as being confined entirely to the art of making reports, fall far short of the classic treatise of the German writer, John Bohn, (*De renunciatione vulnerum,*) in which, among other topics, he discusses infanticide, and declares his disbelief of the hydrostatic test; alleging that the fœtus may breathe in its passage during delivery. In another work, Bohn lays down the twofold duty of a physician in clinical and in forensic medicine, *De officio medici duplici, clinico et forensi,* 1704, and gives rules for the conduct of a medical man in attending the sick, and in delivering evidence before a court of judicature. Frederic Hoffmann, one of the greatest names not only of German but of European medicine, contributed valuable papers on several points of the science, about this period—the end of the seventeenth and beginning of the eighteenth century. Shortly after the publication of Bohn's last-mentioned work, or in 1722, appeared the medico-legal body of jurisprudence, etc., by M. B. Valentini.* This author's rebuke of the medical witnesses of that time are, unhappily, too strictly applicable to those of the present day. Proud of their titles, but yet on these occasions exciting ridicule and losing all the estimation which they once enjoyed. Of a similar character, but inferior merit, was the collection of decisions and observations made by J. F. Zittmann, 1704. The *Systema Jurisprudentiæ Medicæ* of Alberti, in six volumes, dates from Halle, between 1725 and 1747—the former, the year of publication of the first and second volumes; the latter, that in which the sixth volume was brought out. Among the numerous questions elucidated by this laborious writer, may be particularized, those relating to conception and utero-gestation. He is the author of numerous separate treatises and dissertations on subjects of legal medicine. Following the work of Alberti was the *Institutiones Medicinæ Legalis vel Forensis,* by Teichmeyer, in 1722. The merits of these Institutes may be inferred from the fact of their having been made the text for Haller's celebrated lectures on Juridical Medicine, published 1782–1784, after his death. Hebenstreit, in 1753, issued his valuable work, entitled *Anthropologia Forensis,* which is highly prized by jurisconsults. Of later date were the *Institutiones* of Kannegieser, 1777, and the *Conspectus* of Sikora, 1780. To the celebrated surgeon, Antoine Louis, who flourished in the middle and latter part of the last century, is medical jurisprudence largely indebted for illustrating some of the most difficult questions connected

* Corpus juris medico-legale, constans pandectis, novellis et authenticis iatrico forensibus.

with it, both by formal essays and in cases before the courts, in which his opinion was formally invoked. Of these, we may mention more particularly his letters on the certain signs of death; his memoirs on drowned persons, and on the means of distinguishing, in a body found hung, the marks of suicide from those of assassination; on tardy births, etc., which were collected in two volumes, and published in Paris in 1788. Equally luminous, and in advance of all preceding labors of the same kind, were his opinions (consultations) delivered in the cases of Monbailly, Syrven, Calas, Cassagneux, Baronet, etc. etc., which have been preserved in the first forty-three volumes of the *Causes Célèbres.* Petit, a contemporary of Louis, engaged in a controversy with him on the question of protracted pregnancy. About this time, (in 1749,) Beccaria of Bologna sent out his work, *Scriptura Medico-Legalis.* The impulse given to the study of pathological anatomy by another eminent Italian, Morgagni, in the publication of his great work, *De Sedibus et Causis Morborum per Anatomen indagata*, 1762, was productive of a number of happy applications to legal medicine. The doctrine, as it is sometimes called, of infanticide, was brought to maturity by the investigations of Daniel and Ploucquet, on the new methods of ascertaining respiration of a newly-born infant, and by Camper, Metzger, and Portal. Daniel is the author of a Library of Legal Medicine, and of Medical Police, 1784. Ploucquet wrote, at the same time, essays on points illustrating infanticide, and on the diagnostic signs of death. Camper, the celebrated anatomist, contributed two works on subjects of medical jurisprudence: the first, an Anatomico-legal Dissertation on the signs of life and death of newly-born infants; the second, Reflections on Infanticide, together with a plan for a hospital for foundling children; also the causes of infanticide and suicide, accompanied by experiments on the insufflation of the lungs of still-born infants, 1774. Metzger deserves more than a passing notice. His succinct system of Forensic Medicine, published in Koningsburg, 1795, is pronounced by Sprengel to be the best work on the subject; and Marc, a still better judge of its merits, describes it as an excellent systematic treatise. The latter writer speaks favorably also of the collection of consultations by Metzger, many of which relate to the study of mental disease in connection with medical jurisprudence.

The first approach to an entire system of medical jurisprudence in France was by Foderé, toward the close of the eighteenth century—1796. No wise elated by the speedy sale of this work, the author set about maturing his subject; and so intent was he on the faithful performance of his task, that he allowed seventeen years to elapse before he brought out a second edition, which it would be more correct to regard as a new work, cast in a different mould, and both enlarged and improved. Since the beginning of the present century, medical jurisprudence has received large accessions, especially by a closer investigation of the states of mind constituting unsoundness, or insanity, and the application of knowledge thus obtained to determine various civil and criminal questions relating to moral and legal responsibility; and also by a nicer appreciation of the effects of poisons, and of the chemical tests for ascertaining their presence in the human body. The first systematic work in this new period is the posthumous one of Mahon, on Legal Medicine and Medical Police, 1807. Mahon was the professor of these branches, for the teaching of which chairs were first established in France by the revolutionary government. Of a somewhat earlier date, 1800, was the elementary volume of Belloc. Wise and humane reflections on the practice of the science will be found in the treatise of Vigné, 1805. Of a higher character than any work of the kind that had preceded it is the Treatise on Poisons, or *Toxicologie Générale*, by Orfila, which was followed in a few years by his *Leçons de Médecine Legale.* The elaborate work of Devergie, *Médecine Legale, Theorique et Pratique*, carries us still further in the philosophy and varied practical relations of the science. Presenting less of disquisition, and a still larger amount of minute details, is the *Manuel Complet*, the Complete Manual of Legal Medecine, by Briand, which has swelled, in successive editions, from a thin octavo to its present large size. We should be unjust to a great name and to the history of medical jurisprudence were we to omit noticing, in this place, the contributions of Chaussier, who was among the first, in connection with Fourcroy and a few others, to organize a system of medical education under the new circumstances in which France was placed by the revolution. This eminent professor, when yet a young man, awakened attention and excited emulation by a memoir presented to the Academy of Sciences at Dijon, 1789, on the great importance of the study of juridical medicine, and based, we may suppose, on a consultation in the case of an accusation of infanticide two years previously. Chaussier, in addition to the papers which were written in his own name, and which appeared in the form of a collection of consultations, memoirs, and reports, expressed his views through the inaugural theses of some of his pupils. Of this character are those on infanticide; the mode of proceeding in autopsic examinations; spontaneous erosions and perforations of the stomach; ecchymosis, suggillation, contusion and bruises. These four dissertations have been reprinted and bound in one volume, 1819. We derived much instruction from a perusal of them in our earlier study of medical jurisprudence. Worthy of notice, also, is the volume by Capuron, entitled *Médecine Legale relative aux Accouchemens.*

It would be impossible to give any historical notice of French legal medicine without emphatic mention and praise of the invaluable repertory of memoirs, analytical investigations, and practical details on nearly all the topics which go to make up the science, contained in the *Annales d'Hygiène et de Médecine Legale*, during the period of thirty years which has elapsed from the first appearance of that Journal, in 1829, to the present time. Prominent among the many who have enriched its pages, we meet with the familiar names of Bayard, Boys de Loury, Brierre de Boismont, Chambeyron, Chevallier, Devergie, Esquirol, Leuret, Marc, Ollivier (d'Angers,) Orfila, Tardieu, etc. etc.

We shall not, at the moment, name the leading works on medico-legal science by German writers in the present century. They will be spoken of in connection with some of the branches of the general subject. We must not, however, pass over in silence the instructive treatise of Hoffbauer on Legal Medicine, in its relations to the Insane and to Deaf-mutes, 1807. It has been translated into French by Chambeyron, and enriched with notes by Esquirol and Itard.

In reverting to Italian writers on medical jurisprudence we meet with Tortosa, whose Institutions of Forensic Medicine was, at the time of its publication, 1802, regarded as the most elaborate and scientific work of any that had been written in the language on that science. "It is divided into three parts, viz. : 1. *Ecclesiastical* jurisdiction. 2. Subjects relating to the *civil* courts. 3. Those which relate to the *criminal* courts."* Of a recent date are the Introduction to Legal Medicine by Martini, and Legal Medicine by Barzelotti, according to the spirit of the civil and penal laws in vigor in the different governments of Italy. This last is a treatise of the highest order, and may be consulted with advantage as well on the great points involved in the principles of legal medicine everywhere as on the modes of practical procedure in the Italian peninsula. Martini exhibits medical jurisprudence as resting on canon law.

The entire neglect of medical jurisprudence in England, at a time when it had engaged the attention of so many able writers in France, Germany, and Italy, may be inferred when we say, that, with the exception of the small and imperfect production of Farr, no work, with any pretension to a systematic character, made its appearance in that country before the beginning of the present century. Farr's small volume, published in 1787, bore the title of "Elements of Medical Jurisprudence." Our edition is the second, which was brought out in 1814, a circumstance of itself proving the limited views and imperfect knowledge of the science at this time, when such a tiny duodecimo, of only 139 pages, was all that was offered to meet the wants of English readers. It is, after all, as the author admits in his preface, little more than an abstract from the *Elementa Medicinæ*

---

* Paris and Fonblanque.

of Faselius. The next, in succession, was a "Treatise on Medical Police," by Dr. Robertson, in two volumes, published in 1808. Dr. Bartley, of Bristol, is the author of a "Treatise on Forensic Medicine," than which it is impossible to conceive any production more meagre or imperfect. Dr. Male is undoubtedly entitled to the grateful notice of the medical historian as the author of the first respectable English book on Forensic Medicine. Our copy is of the second edition, 1818, and consists of 278 pages small octavo, dimensions which would fall far short of the requirements of the present day. Of a more comprehensive and instructive character than any which preceded it, is the "Principles of Forensic Medicine, systematically arranged and applied to British Practice," by John Gordon Smith, M.D., 1821. Useful information is communicated by the same author in "Hints on the Examination of Medical Witnesses," 1829. "In addition to the above writings, we may record the 'Medical Ethics' of Dr. Percival, which, although not intended, nor indeed calculated for practical instruction, contains some interesting allusions to our subject. Nor must we omit to enumerate the several valuable monographs with which different English physicians have sought to advance the progress of medico-legal inquiry; as, for instance, the celebrated paper of Dr. Hunter 'On the Uncertainty of the Signs of Murder in the case of Bastard Children,' Dr. Haslam's intelligent and judicious essay 'On Medical Jurisprudence as it relates to Insanity, according to the Law of England,' and Dr. Hutchinson's laborious 'Dissertation on Infanticide.'"* Two years after the publication of Dr. Smith's work, Dr. Paris and Mr. Fonblanque, a physician and a lawyer, brought out their joint contributions in a formal and elaborate shape, under the title of "Medical Jurisprudence." The authors display copious reading and minute research, and some of the leading topics are elucidated by their labors, but the production lacks unity and method, and contains irrelevant matter, or such at least as often calls off the attention of the reader.

The first and for a long time the only chair of Medical Jurisprudence in Great Britain, was endowed in the University of Edinburgh in the year 1806. The first incumbent was Dr. Andrew Duncan, Jr., whose father had been mainly instrumental in directing the attention of the public and the government to the importance of the subject. Of the gross ignorance of its simplest principles, and indeed of the whole science, by educated men in England, at this time, some idea may be formed from the observations

---

* Paris and Fonblanque, Medical Jurisprudence. We are indebted to these writers for a notice of English works on the subject to the date of the publication of their own, 1823, as well as for some of the bibliographical references in the preceding history. Our chief obligations in this way are due, however, to Foderé's *Introduction* and to Wildberg's *Bibliotheca Medicinæ Publicæ*.

made by Mr. Percival and Mr. Canning, in the House of Commons, in their strictures on the late or Fox ministry. We must premise that there are chairs in the University, thè creation and filling of which rests with the crown, and the incumbents are hence called Regius professors. Mr. Percival, afterwards prime minister, and who had filled the offices of solicitor-general and attorney-general, said, in the course of the debate, that "he should not dwell in detail upon all the acts of the late administration, but he confessed himself at a loss to understand what they could mean by the appointment of a Professor of Medical Jurisprudence. He acknowledged that he was ignorant of the duty of that professor, and could not comprehend what was meant by the science he professed." Mr. Canning, in the same debate, assuming a tone which might have been justified by the creation of a professorship of Astrology and Divination, "could alone account for such a nomination by supposing that, after some long debate, in the swell of insolence, and to show how far they could go, they had said, 'we will show them what we can do—we will create a Professor of Medical Jurisprudence.'" We are at the moment restrained from strictures on this gross ignorance, so publicly, and, as it were, ostentatiously displayed by men in high places, when we reflect that even now, after the flood of light shed upon the science of medical jurisprudence in all its divisions and ramifications during the last half century, the trustees and faculties of most of our medical colleges seem to be still groping their way, and quite as much at a loss to guess the meaning of the subject as were Messrs. Percival and Canning. We are left to draw this inference from the total want of measures for having this most important branch taught from a separate chair, or indeed taught at all.

Resuming our sketch of the progress of medical jurisprudence in England, we next meet with the elaborate and truly scientific work of Dr. Christison, on Poisons, to which currency was given in this country by the publication of the fourth edition, 1844.* The first edition appeared in 1829. A systematic work on medico-juridical science was begun by Joseph Chitty, an eminent pleader, and author of different legal treatises. The first and only part which ever saw the light was published in 1834. Its title, a Practical Treatise on Medical Jurisprudence, etc., etc., is a decided misnomer, as the volume is, in fact, a summary of anatomy, physiology and hygiene, with, here and there, some applications to medical jurisprudence. The author had studied medicine before law, and seems, in consequence, to have been unduly desirous of impressing his associates at the bar with a high idea of his knowledge of the literature of the profession to which he had first applied himself. The work was republished in

* In Bell's Select Medical Library. 1845.

this country, with notes. Ryan's small volume may be dismissed in a few words. Its American editor, Dr. R. E. Griffith, had higher requirements for a task of this kind than his author. Dr. Traill, the successor to Dr. Duncan in the chair of medical jurisprudence in the University of Edinburgh, is the author of a succinct and well-written notice of the general principles of the science—too brief for a systematic work, but good as a text-book. It also has been republished in the United States, with notes, 1841. In coming down to the present date, we can speak with confident praise of works on medical jurisprudence which approximate to a higher standard than was ever previously thought of in England. A reference to the treatises of Dr. Alfred S. Taylor and of Dr. W. B. Guy will fully confirm this assertion; and, we may add, that they have been enriched with notes and commentaries by their able American editors: Dr. Edward Hartshorne rendering service in this way to the former, and Dr. Chas. A. Lee to the latter of these two authors. Dr. Taylor's work on Poisons is also a valuable addition to the science of legal medicine. In the same line of successful illustration of separate points of the main subject, reference may be made to Watson's Medico-legal Treatise on Homicide, and Gavin's recent work on Feigned Diseases.

The principle of compensation prevails in the affairs of man to an extent not adequately appreciated, and in the matter of medical jurisprudence on American ground, while we have great cause to repine at the neglect to diffuse a knowledge of it by oral instruction from chairs in our medical and law schools, we ought to be thankful that it is not only saved from oblivion, but is kept alive, and, in a measure, watched with some interest by numerous volunteers from the great body of the profession; thus adding one to the many examples of the popular mind giving expression to wants and devising means for their gratification, in advance of the slow and hesitating steps of the delegated organs of public opinion and of the authorities who are supposed to be its exponents. American literature may plume itself on some works on medical jurisprudence of established character and undoubted reputation. First in the order of appearance, and in copiousness of matter and minuteness of detail on every point which can come up for discussion in our courts, is the voluminous treatise of the two Becks, Drs. Theodric Romeyn and John B., of whom the first is the designer and chief author. This work, in its successive editions, has attained such a magnitude and exhibits such a variety in its several parts as to approach to the nature of an encyclopedia. In this respect, indeed, the reader may sometimes labor under the rare inconvenience of an *embarras des richesses;* a fullness and an expansion which would interfere with a ready grasp and immediate comprehension of the point at issue. But the great, the essential requisite is fulfilled in the labors of these writers, viz.: the presence of all that is wanting, fairly and honestly, and, for the most part, artistically arranged. After all, who would not prefer abundance, even to redundancy, a comely, albeit somewhat obese form, to one with a meagre and sharp outline? This great work, modestly called "Elements of Medical Jurisprudence," has been repeatedly published in England, under the able editorial supervision of Doctors Dunlop and Durwall, and its value is warmly appreciated by the medical jurists on the continent of Europe. Doctor Romeyn Beck, in the introduction, bears grateful testimony to the merits of his preceptor, Dr. James S. Stringham, who was the first to deliver a course of lectures on medical jurisprudence on this side of the Atlantic. In 1804 this gentleman, then Professor of Chemistry in Columbia College, N.Y., voluntarily added to his regular course one on legal medicine. In 1813 Dr. Stringham was appointed Professor of Medical Jurisprudence in the College of Physicians and Surgeons of New York; but his failing health interfered with the active discharge of the duties of this new chair, and his death, in 1817, was a final blow to further labors in this line. Dr. R. Beck was appointed to lecture on Medical Jurisprudence in the Western Medical College of New York; and not long after Dr. Walter Channing was elected to perform a similar duty, in addition to that of his professorship of Midwifery, in Harvard University. Lectures on Medical Jurisprudence were delivered by Drs. Williams, of Berkshire Medical Institute, and Hale, of Boston, in the winter of 1822. The eloquent and impressive recommendation of Dr. Rush, in an introductory lecture in the University of Pennsylvania, 1810, in favor of the science, was not followed by any regularly devised plan for conveying instruction in it, nor have we heard of his urging on the trustees of the school the creation of a chair of medical jurisprudence. Possibly it was under an inspiration of the sort that Dr. Caldwell delivered a course of lectures on the subject in the winter of 1812–13. We are not apprised of these having been repeated, and we must infer that the eloquent lecturer failed to procure an auditory to encourage further efforts in that line. Judge Cooper, a friend and countryman of Priestley, after leaving the bench, resided for a time in Philadelphia, and was connected with the University of Pennsylvania as Professor of Chemistry and Mineralogy applied to the arts.* During this period he edited "Tracts on Medical Jurisprudence, including Farr's Elements, Dease's Remarks, Male's Epitome, and Haslam's Treatise on Insanity, together with a Digest of the Laws relating to Insanity and Nuisance, and an Appendix, containing Erskine's speech for James

* Judge Cooper had filled a similar post in Dickinson College, Carlisle, Pa. He was ultimately connected with Columbia College, S. C., in which institution he was at first Professor of Chemistry, and afterwards President.

Hadfield, indicted for shooting at the King; an Abstract of a Report of the Trial of Abraham Kessler, indicted for poisoning his wife with white arsenic and laudanum; and a Memoir on the Chromate of Potash as a Test for detecting Arsenic, Copper and Corrosive Sublimate, 1819." The opening remark of the editor, in his preface, that "the study of Medical Jurisprudence is much more attended to on the continent of Europe than in Great Britain or in this country, where it has by no means received the attention due to its importance," is still applicable to both countries. We quote another sentence, which occurs toward the close of the preface, and which indicates wants not yet supplied in our medical schools. "In the present state of general knowledge, juries will not be satisfied to receive from a medical witness the very slight information he may have brought away from the place where he received his medical education; and unless he attaches more importance to Medical Chemistry, as he grows older, than he is taught to connect with it here, he incurs the hazard of finding more knowledge of the subject among those who are not of the profession than he professes himself." The next attempt to diffuse some elementary knowledge of the subject was made by Doctor Bell, who taught every year, for a period of eighteen years, in the Philadelphia Medical Institute, Medical Jurisprudence in addition to the Institutes of Medicine. The interest manifested in the lectures on the former branch by the class gave assurance of success to any subsequent attempt in the same line, if made on a larger scale and with more scientific appliances than were then at the disposal of the lecturer. Reference has been made in a preceding page to the creditable additions to English works on Medical Jurisprudence by American editors. We have now to speak of a production which is at once among the latest and the best on the science. It is "A Treatise on Medical Jurisprudence, by Francis Wharton, Esq., and Moreton Stillé, M.D., 1855,"—in the composition of which legal and medical acumen and lore have been happily combined.

We adverted, in a preceding page, to the diversified applications of the study of insanity to medical jurisprudence. The subject has enlisted the attention of some of the ablest writers during the last forty years, and it may be said to have given origin to a new branch of literature. The courts of law and the people generally had been willing to receive the plea of insanity as a bar to punishment for acts, which, if committed in a sane state of mind, would necessarily be regarded and treated as criminal. But both the courts and the people were startled at the first annunciation made on medical authority, as the result of medical observation, that a similar exemption from legal penalties may be rightfully claimed by individuals who are only partially insane, and whose mental faculties are manifested in the ordinary and natural manner, with the exception of the disordered faculty or propensity. This morbid condition of mind indicated by Pinel,* and to which Esquirol † was the first to affix the term *monomania,* has been acknowledged and commented on by their successors in the field of clinical and philosophical observations on insanity, or unsoundness of mind. The medico-legal relations of this view of the subject were seen at once, and have led to much discussion, which has sometimes been conducted with a heat bordering on acrimony. Our object now is not to discuss its merits, but merely to refer briefly to its literature, as developed in the pages of Georget,[1] Marc,[2] Briere de Boismont,[3] Leuret,[4] Calmeil,[5] Prichard,[6] Conolly,[7] Ray,[8] Pagan,[9] Simpson, (a lawyer,)[10] Clarus,[11] Hoffbauer,[12] Masius,[13] Haindorf,[14] Conradi,[15] Reil,[16] Wildberg,[17] Schulze,[18] Heinroth,[19] and most of the German medical legists, and also Feuchtersleben.[20] Among these we would make special mention, without undue national bias, of the luminous treatise of Dr. Ray, on the "Medical Jurisprudence of Insanity"—a work not only creditable to American medical literature, but one which has taken a high stand among foreign authorities. It preceded, by a period of two years, an English treatise with the same title, by Dr. Pagan.

Our readers, we hope, will not think that the time has been unprofitably spent in taking a retrospective view of the great field of medical jurispru-

---

* Alienation Mentale.

† Des Maladies Mentales In the Abridged Translation by D. E. K Hunt, that portion of the work on medico-legal relations of Insanity is unfortunately omitted.

1 Sur la Folie.

2 De la Folie considerée dans ses Rapports avec les Questions Medico-Judiciaires.

3 Des Hallucinations, ou Histoire raisonnée des Apparitions, etc.

4 Du Traitement Moral de la Folie. Also Ann. Hyg. et de Med. Leg. *passim.*

5 De la Folie considerée sous le Point de Vue, Pathologique, Philosophique, Historique et Judiciaire, etc.

6 A Treatise on Insanity and other Disorders affecting the Mind.

7 Inquiry concerning the Indications of Insanity. Also "A Remonstrance."

8 Medical Jurisprudence of Insanity. 9 Ibid.

10 Criminal Jurisprudence considered in relation to Cerebral Organization.

11 Henke's Journal für die Staatsarz neilkunde. 1824.

12 Médecine Legale, relative aux Alienés et Sourds Muets (Trad. en Franç.) 1827.

13 Handbuch der gericht. Arzneiw. 1823.

14 Versuch einer Pathologie und Therapie der Geistes Krankheiten.

15 Commentatio de Mania sine Delirio. 1827.

16 Rhapsodien über die Anwendung der psychischen Kurmethode auf Geisteszerrüttungen. 1803.

17 Lehrbuch de Gerichtf. Arzneiw. 1824.

18 Psychische Anthropologie.

19 System der Psychisch-gerichtl. Medicin. 1825.

20 The Principles of Medical Psychology. Translated from the German by H. E. Lloyd, for the Sydenham Society.

dence, and of those who have acted a prominent part in it. We now pass on to a notice of the work of M. Briand. In an introduction of sixty pages, the author details the duty of a medical legist before the tribunals, both in criminal and civil cases, as laid down in the Code Napoleon. He discusses the following points: 1. What is the course to be followed in the inquiry and prosecution for crimes and misdemeanors? 2. In whom rests the authority to require the assistance of professional men? and are there cases in which these latter may decline to comply with the requisition made on them? 3. If among professional men there be any on whom the law more particularly relies. 4. What are the necessary preliminaries in all cases of *expertise*, and the general steps to be taken by the expert on the execution of the mission confided to him? 5. What are the rules to be followed in the preparation of reports, consultations, and certificates? 6. In what cases are physicians or surgeons responsible for the result of cases in their practice? 7. Finally, what are the honoraria allowed to physicians, surgeons, etc., in any case in which their assistance is invoked by the criminal courts? The consideration of these questions involves the whole subject of medical evidence and of medical responsibility, and constitutes an appropriate introduction to any professedly systematic work. Orfila and Devergie begin in the same way. Of the English authors, Paris and Fonblanque, after a sketch of the progress of the science, in the shape of an introduction, and a historical *hors d'œuvre* on the charter and powers of the Colleges of Physicians and Surgeons and of the Society of Apothecaries, treat of medical liabilities and exemptions, and of medical evidence generally. Gordon Smith, as we have seen, deems the question of sufficient importance to make it the subject of a separate dissertation. Dr. Guy begins his volume by a well-written chapter on medical evidence, to which succeeds one on personal identity; on the other hand, Dr. Taylor plunges at once, *in medias res*, by discoursing on poisons. The first chapter of Foderé's work, following his learned introduction, is on "Ages," the same as that discussed by Orfila. Beck opens with "Feigned Diseases," and Wharton and Stillé with "Unsoundness of Mind." Preference must, we think, be given to the initial course pursued by M. Briand and other French writers on legal medicine, and by Guy of the English ones. It is very desirable that a medical man, who may be summoned to give either oral or written evidence before a court, should know the ground on which he stands—the customs and laws by which he is fenced in, as well as be familiar with the purely scientific matter of which he is expected to speak understandingly, and with a certain degree of authority. Before we proceed, however, to investigate this ground, which we can hardly hope to reach on the present occasion, we shall complete our synopsis of M. Briand's work. We must say, at the setting out on this part of our task, that there is no natural classification of the subjects which properly make up the science of medical jurisprudence. Two writers may start from opposite points, and each claim his own as the most appropriate beginning; as, for instance, Foderé and Orfila, in choosing the "ages" for their initial theme, while Devergie begins with a discussion on "death and its signs." Among the first questions asked are those of the age of an individual who is the subject of a medico-legal inquiry, with a view of determining the privileges, exemptions, and responsibilities which belong to it as well as the point of personal identity—as in the cases of supposititious children, accusations of crime, claims to the possession of property, etc. Looking, on the other hand, at medico-legal proceedings in a criminal point of view, and regard being had to the importance of ascertaining whether, in the case of a person found dead, his death was the result of internal disease or of external violence, of causes purely accidental, or owing to criminal intention, the questions of the various modes in which it may take place, as from organic disease previously known or latent, or from hanging or drowning, and of its determining causes, as well as the period that had elapsed subsequent to its occurrence, assume a primary importance, such as might well justify Devergie for giving to a consideration of them nine chapters of the first volume of his valuable treatise.

M. Briand divides his work into two parts, viz.: I. Legal Medicine; II. Legal Chemistry. The former is subdivided into four sections; in the first of which, he treats of attempts on modesty and the reproduction of the species; in the second, of attempts against health and life; in the third, of mental affections; and in the fourth, of questions of identity, feigned diseases, and diseases which exempt from military duty. In the second part, of which M. Gaultier de Claubry takes charge, we read, in the first chapter, of an investigation of poisons; in the second, of substances which although not poisonous come within the quest of legal chemistry. This latter branch of the inquiry turns on the use and application of the microscope in a great variety of circumstances in which minute inspection of the physical characters of the blood and of blood spots, and of other fluids of the body, is of the last importance. Following this chapter are examples of reports on different cases of criminal accusation, and on microscopical examinations. To these succeed a statement of the laws, decrees, and ordonnances relating to medicine and pharmacy, and commentaries on these laws. This portion of the volume, together with all the jurisprudential matter in the body of it, at the beginning of each chapter, has been contributed by M. Chaudé.

Returning now to the first section of the work of M. Briand, with its somewhat indefinite heading, we find that under attempts against modesty he discusses the important questions of rape, and in that which relates to the reproduction of the species he treats of the causes preventive of marriage; then of pregnancy, abortion, and child-birth; medico-legal investigations relating to delivery, premature and tardy births; the life and viability of newly-born infants; concealment, simulation, substitution and exposure of a new-born child; infanticide; questions growing out of the presumption of infanticide; the course to be pursued by a medical man called to make a report on a case of infanticide.

The second section, being that on attempts against health and life, furnishes three chapters. The first of these is on blows, wounds, and homicide by blows or wounds; also on suicide and duel. It opens with an account of the legislation and jurisprudence touching homicide, blows and wounds; and discusses the questions, whether suicide be a crime punishable by law; and whether the death is the result of a suicide or of a homicide. Wounds are reviewed in reference to the mode of lesion. A separate article is allotted to burns. Wounds are noted according to the part of the body or the organ in which they are seated. Cicatrices are studied in this connection. A juridical examination of wounds is laid down, and in a succeeding article is a notice of stains of blood and cerebral substance, similarly examined. A judicial examination of the body of a person whose death has been the result of suspected violence, forms the subject of a long article, divided into many subheads, such as on the method of making the autopsy; spontaneous lesions which may cause sudden death and give rise to the suspicion of its having been the result of violence; how to distinguish, in a dead body, the lesions inflicted during life from those made after death; how long since has the death occurred; the signs of real death; burials, and disinterments.

The second chapter deals with asphyxia, under the varieties caused by noxious gases, drowning, hanging, strangulation, and suffocation. Chapter third contains an account of homicide by poisoning, including an inquiry into the poisons most generally employed, and the mode of their introduction into the organism; and also into the symptoms and lesions to which they give rise. The first class specified is that of irritant poisons, subdivided into mineral, vegetable, and animal. The second, or the narcotic class, is the next described. The third, or narcotico-acrid, includes several groups, in the first of which figure squills, aconite, hellebore, colchicum, belladonna, stramonium, tobacco and nicotine, digitalis, hemlock, conicine, and the laurel-cherry. In the second, are seen nux vomica and faba St. Ignatii, strychnine and brucine; in the third, camphor and cocculus Indicus; in the fourth, mushrooms; in the fifth, ergot of rye, darnel, and the ranunculaceæ; in the sixth, alcohol and alcoholic liquors; and in the seventh and last group, ether and chloroform. The fourth class gives us septic poisons, such as the sulphydric acid gas, the mephitic air of sewers, etc., and tainted food. The third article of this chapter is devoted to the subject of the autopsy of persons who have been poisoned, and to the necessary investigation for determining the fact of poisoning. Among the questions coming up in this category are those relating to the nature of the soil in which the body was interred, and to the possibility of the arsenic in the soil penetrating into this body; and thus induce a belief of our having to do with a case of poisoning: or the converse supposition, that the arsenic contained in a body which has been poisoned may have been dissolved and carried away by the water which filtrates through the surrounding soil, so as to leave no trace of poisoning. When analyses are necessary they should only be undertaken by chemists qualified for the duty.

The third section of the work before us is on "mental affections." As in the plan of the preceding sections, this one opens with a view of the legislation and judicial decisions on this interesting theme. Then we read of the more marked deviations from the healthy standard of mental soundness, viz.: idiotism and imbecility; and mental alienation, or madness or insanity properly so called, in its varieties of dementia, mania, and monomania. The causes of insanity, as well as the symptoms and progress of the disease, are exhibited. The question, does monomania exclude culpability? is discussed. The passions constitute the subject of an article in which it is asked: Does the bewilderment caused by the passions exclude moral liberty? Next is investigated the influence of certain physiological and pathological states on moral liberty, viz.: somnambulism, the state intermediate between sleeping and waking, febrile delirium, acute delirium, drunkenness, delirium tremens, epilepsy and hysteria, deaf-mutism. The fourth, the heading of which has been already given, opens with the question of identity, and the signs by which it can be ascertained; first in the living, and then in the dead subject. Feigned diseases make up the matter of the second chapter, and the diseases which exempt from military duty the third one.

The Second Part of the work, or Legal Chemistry, first chapter, begins with an investigation of poisons, conducted as follows: The preservation of the matters collected in a case of poisoning; the procuring of vessels, apparatus, and other objects necessary for the search after the poison; the tests and the various substances employed by the experts or regularly appointed medical or scientific legists. In article fourth are passed in review poisonous substances met with in nature. Of these we find certain metals and the simpler metallic preparations, such as antimony and its sulphurets, metallic arsenic and fly powder, copper and its compounds, mercury in its metallic state; and also an elementary substance of a different order—iodine. Next are specified colorless or white solid substances; dry, metallic compounds of antimony, silver, bismuth, tin, mercury, lead, and zinc; acids, including the arsenious, arsenic, and the arsenite and arseniate of potash, oxalic acid, and the binoxalate of potash; alkaline and earthy bases and their salts—ammonia, potassa, soda, and their combinations with acids; organic alkalies, fixed, volatile, and gaseous. The first are, atropine, or elaterine, brucine, codeine, morphine, saponine, strychnine, veratrine; the second include conine or conicine, nicotine, petonine, and picoline. But we find that our remaining space will not admit of an enumeration of the numerous substances spoken of in this chapter, which are somewhat arbitrarily classified according to their color. The sulphates and nitrates of copper, for example, come under the head of solids of a blue color; and sulphuret of arsenic, (orpiment,) certain chlorides, and the iodide of gold and turpeth mineral ranging among the yellow solid substances. A search for poisons in suspected matters, as in the products of vomiting, etc., is described with considerable minuteness: it begins for simple bodies, then for acids, alkalies, and their salts, metallic poisons, arsenic. The nature of deleterious gases is then examined, and the chapter closes by inquiries into the anæsthetics.

The chapter on the tests furnished by the microscope, in the inspection of the blood and blood stains, and of the marks left by other animal fluids, is full and instructive. Another article exhibits the means of ascertaining the nature and the color of the hair and the beard. Fire-arms is the next theme, which embraces an inquiry into the means of determining the period that has elapsed since a fire-arm was discharged. After this we find a description of the processes for detecting the prints left on the ground by tne feet, or by carriage wheels, etc.; also for examining the ashes in a particular spot, with a view of discovering the remains of a dead body. Forged writing, and counterfeit coins, and imitations of the precious metals, are the subject of an article in this rich chapter, which terminates with a detail of various matters for the exercise of the science and ingenuity of an expert, as they come before the minor courts, such as, for instance, alteration and adulteration of flour, bread, wines, vinegar, and milk.

Civil cases, ranking properly under the head of nuisances, are examined and detailed in their medico-legal bearings. Some of the cases are singular enough. The owner of a cow-house brought suit against a sugar refiner, on the plea that the water of the establishment of the defendant affected his (the prosecutor's) well. On examination, it was found that the infected water had a very decided odor of cow's urine, which became still more evident by evaporation to a syrupy consistence. By further treatment, oxalate of lime and urea were obtained. It was evident, therefore, that the offensive water of the well was caused by infiltrations from the cow-house into the yard of the complainant, and not from the water of his neighbor's premises. Many questions come up in business dealings which can only be adjudicated after the testimony borne by a chemist who has made the requisite examination; as, for example, in the case of the owner of a house who had employed a person to repair it, the painting was to be in oil, and of three coats; but it was alleged by the landlord that only one coat of oil had been put on over a sized coat. M. Gaultier de Claubry, the expert, on this occasion scraped off the painting from a certain number of points, and placed each specimen in a paper duly labeled. Each portion of the painting was then treated by heat with ether to dissolve the oil, which was obtained by spontaneous evaporation; the residue was boiled with water, which extracted the gelatine, and this was afterwards obtained by evaporation. The new insoluble residuum was composed of carbonate of lime. The fact of the sizing being proved, it would be easy to tell with some certainty whether one or two coats of this size have been put on. This knowledge is derivable from the proportionate quantities of oil and gelatine.

In further proof of the necessity of admitting chemistry as a branch, under the title of "legal medicine," but still related to medical jurisprudence in its larger signification, M. Gaultier de Claubry adduces instances of the aid furnished from this source in various questions relating to the public health, but which do not strictly come under medical supervision. We may repeat after him some of these. In hearing of the escape of the foul waters from the cistern of a gasometer which was not sufficiently tight, and their infiltration into places around, the question is, not to ascertain their effect on the animal economy, but to point out the remedy for this condition of things. When a manufactory of oxalic acid diffuses nitrous vapors through the atmosphere, which are known to be hurtful to the neighborhood, the mode in which their condensation is to be effected is the sole question. A manufactory of fulminating mercury and priming is established at a certain spot: the conditions to be fulfilled, so that the neighborhood shall be put to the least inconvenience from it, are the points for examination. Particular processes are had recourse to for the emptying of privies and the removal of their contents. Here there is no question respecting the action on the animal economy of the gases given out from these receptacles and their contents; it is only to ascertain whether the means proposed for the purpose obviate the resulting inconveniences. The last example coming under the present head presents points of great practical value, in relation to the deleterious changes which may take place in the staff of life. All at once, in the year 1840, and without any known cause, the bread of the military bakeries, for the soldiers of the garrison of Paris, was found to be covered with red spots of a decided hue. This bread soon emitted a disagreeable, and even sickening odor, and it was unfit to be sent out for consumption. But few examples of this nature were met with in the civil bakeries. The change, it was ascertained, was owing to a mushroom of the genus *oïdium*. On examining the circumstances under which it was found, the author was led to search in the flour itself for the sporules of the mushroom, and he found them. The cause of the alteration being known, the next point was to remove or destroy it; and accordingly various means presented themselves for the purpose, viz., the mixture of the inferior flour with that of a superior quality; the preparation of harder biscuit, longer worked; baking in an oven at a lower heat; exposure of the baked bread in the open air, in place of immediately stowing it away as had been the ordinary practice. By the employment of these means the deleterious change was prevented.* We are sorry that occasion is given to us for repeating the brief remark of M. Gaultier de Claubry, which follows the preceding statement. It is, that "subsequently a commission appointed by the minister of war pointed out the same results, without even mentioning my publication on the subject."

The latter portion of the rich volume before us consists of examples of reports on different cases of actual or alleged crimes, for the proof or detection of which, as far as relates to injury to the health and to loss of life, a medical jurist or a chemical expert was consulted; then of the laws and regulations relating to medicine and pharmacy, and the commentaries on these laws. In availing ourselves of the facts and reasonings in these matters, we ought to connect them with the details furnished in the Introduction, relating to the initiatory steps and subsequent course to be taken by the constituted authorities and law officers, for procuring the co-operation of medical men. We could not well undertake to follow our author in these interesting topics, without engaging to a certain extent in a discussion of the modes of procedure carried out in different countries in medico-legal investigations. The subject is more than merely curious; it involves questions of great moment, and merits a larger treatment at our hands than would be possible so near the conclusion of a paper which has already reached its originally assigned limits. It would be necessary, were we to open out the subject, to begin with a comparison of the crude proceedings according to the law of coroner, as received in England and in this country, with the system of expertise in France and that of the Supreme Medical and Sanitary Council, and the *Staats Physicus or kreis-Physicus*, in most parts of Germany. We

---

* Annales d'Hygiène et de Médecine Legale, xxxiv. 347.

cannot, however, close without a few practical lessons, equivalent to clinical instruction, when teaching the theory and practice of medicine, as we find them in the shape of reports contained in M. Briand's volume.

Our first selection will be of a Report on a case of hanging; "suspension," as it is termed in the French idiom:—

We, the undersigned,* in order to ascertain the kind of death of M. H., who is said to have hanged himself this very day, at five o'clock in the morning, having arrived at said spot, where we found the commissary of the police, were introduced into a dimly-lighted room, on the ground floor, in which there was hung from a bar of iron, raised about 2·60 metres (8 feet 6 inches) from the floor, a body which we were told was that of M. H.

A smooth rope, of the thickness of a finger, made a running noose tightly drawn round the neck. The tie of this rope was placed under the right side of the chin, the head was thrown far back, and inclined in such a way that the occiput was brought near to the left shoulder, and the head was turned upward and to the right side.

The posture of the body and the degree of constriction of the running noose are proofs that this individual had cast himself off with force from a chair which was found upset at another end of the room.

After having assured ourselves, by an examination of the room, that there was nothing to furnish any pertinent evidence, we cut the rope near the end which was tied to the iron bar, and had the body removed to a better lighted place, in order to proceed to a detailed examination.

I. The face and lips, instead of being swelled and of a blue or violet color, as they are for the most part in persons who have been hanged, were, the first pale, and the last colorless.

II. The eyelids of the left eye were closed, and those of the right eye half open; the pupils were greatly dilated; the eyes neither injected nor prominent.

III. The tongue was thrown backward and drawn into the pharynx.

IV. A little frothy mucus issued from the nose and mouth when the thorax was compressed.

V. After having removed the rope, we observed a horizontal sulcus or furrow, nearly circular, and about 16 or 17 millimetres in depth, and the skin of this part of a yellowish hue and dry, like parchment.

VI. The hyoid bone was fractured, and thrown far back by the pressure of the rope.

VII. There was no ecchymosis either above or below the furrow, nor in the super or sub-hyoidean muscles; but after having removed the integuments of the posterior part of the neck and trapezius muscle, which was entire, we found extensive infiltration in the splenius, the great and smaller complexus, the transverse, and the spinal transverse muscles: the cervical ligaments were entire, but

* In making a judiciary report, the expert, or regularly enlisted medical jurist, or other person summoned on the occasion, gives his prename and surname, and his titles and professional position..

stretched; the cervical vertebræ were neither fractured nor at a greater distance from one another.

VIII. The integuments of the cranium, the bones, and the organs contained in this cavity, showed not the slightest trace of lesion. The cerebral substance was only a little redder than in the natural state.

IX. The larynx and the trachea contained a bloody mucus. The lungs were free from all adhesions, and were very crepitant; they were gorged with dark and fluid blood, as were also the right cavities of the heart; the left cavities of this organ were entirely empty.

X. The abdominal viscera were in their normal state, with the exception of the stomach, which exhaled a strong alcoholic odor: its mucous membrane was inflamed, doubtless owing to the abuse of strong drinks, to which, we were assured, the deceased had been for a long time addicted.

XI. The penis was in a state of erection, and that part of the shirt in contact with this organ exhibited moist and yellowish spots, spots caused by the semen which had been ejaculated, as evinced by the odor and as we have already shown. (Page 769, *et seq.*)*

CONCLUSIONS.—Although the body subjected to our examination has neither presented that apoplectic state which authors have specified among the signs of death by hanging, nor that projection of the tongue, nor that prominence of the eye which are often remarked in such cases, the absence of these signs is not a sufficient reason for our doubting that the death was the result of hanging. The fracture and retroversion of the hyoid bone seem to us to account both for the retraction of the tongue and the presence of less blood in the encephalic vessels, which were closely compressed. We believe, consequently, that the circular mark, and the numerous ecchymoses, of the presence of which we assured ourselves, (VII.,) the quantity of dark and fluid blood in the lungs and in the right cavities of the heart, the vacuity of the left cavities of this last-mentioned organ, the erection of the penis and the ejaculation of semen, furnish proof that the individual submitted to our examination did really perish by hanging.

In proof of which, etc., etc.

The only criticism we have to offer at the moment on the above report is on the use of the word inflamed. The term inflammation is an abstract one; the phenomena constituting that state do not present themselves to all in the same light. It were better, in such cases, to specify the appearance of an organ and then add, "as if inflamed." We may beg the use of the word in pathological inquiries, but it is hardly admissible in circumstances of pure description, where we wish to convey to the mind of another person—and he, perhaps, not initiated in the language of science —a definite idea of actual appearance or occurrence. In another "report" on a case of death from hanging, no ecchymosis was met with under the sulcus caused by the rope; nor was there any laceration, or solution of continuity of the hyoid bone, or the larynx or trachea.

---

* The details on this point will be given in the next "Report."

Our next selection is of "Reports on a case of Rape, (*defloration*,) with violence."

First Report—Examination of the complainant:—

We, the undersigned, N., doctor of medicine of the Faculty of Paris, living in —— street, on the requisition of M., the procureur imperial, and after having taken an oath before this magistrate to make our report and give our opinion on our honor and to the best of our belief, went this day, Tuesday, the 28th of October, 185–, in —— street, number ——, accompanied by the commissary of police of the quarter of ——, for the purpose of visiting the daughter of Mr. M., who is represented to have been ravished and deflowered on Sunday, the 26th, at eight o'clock in the morning.

Mr. M. showed us his child, aged twelve years, who complained of sharp pains in the genital organs, the thighs, and lumbar region. He told us that, on Sunday morning, Mr. E., aged twenty-five years, had enticed her into a room in the story below, and had subjected her to violence, in spite of her resistance; that his child had not been affected previously with any catarrhal complaint; that she had not yet menstruated, but that she enjoyed, habitually, good health, as seemed to be evinced by her appearance.

M. showed us the shift which she wore at the time when the violence on her person was committed, and we noticed on it stains of different substances which we reserve for examination.

Proceeding at first to the inspection of the young girl, we ascertained that she is of a middle height, of ordinary plumpness for her age, and of a good constitution, and that there is nothing in her physiognomy to indicate vicious habits.

The sexual organs, regularly formed, exhibit the following appearances:—

I. The external labiæ, slightly apart from one another, are red and swollen on their internal surface; the inner labiæ are much tumefied, very red, and covered with purulent mucus of a yellowish-white color, which flows more abundantly from the vaginal orifice when we press on the perineum, but not at all from the urethra.

II. The clitoris is but little developed, and the fourchette is entire.

III. The membrane of the hymen is torn from above downward, and the torn edges form on each side of the entrance into the vagina, which is sensibly enlarged, a projecting fold, with borders unequal, red, swollen, and slightly excoriated.

IV. We observe, besides, at the inferior and internal part of the thighs, and on the right forearm, contusions which seem to us to have been made two or three days previously, as were the injuries done to the sexual organs.

From these observations we believe that we may infer:—

1. That the young M. has been recently deflowered. (III.)

2. That the introduction of a virile member, or some body or another, has taken place, in spite of the lively resistance on the part of the complainant. (II.)

3. The state of health, the general habit of body, and regular conformation of the sexual organs, render improbable the existence of a catarrhal affection and of vicious practices; the discharge from these parts appears to us to be only attributable to violences of such as this young girl alleges she was the victim.

These presumptions of rape, already so weighty, resulting from the state of the girl M., will acquire a high degree of certainty if an examination of the stains on the shift show that they are caused by semen.

Accordingly, the said shift has been made up into a package, and fastened with cord, to which has been affixed the seal of the commissary of police, in order that a chemical examination may be made of the stained spots.

We certify to this report being made in sincerity and truth.

At Paris, day, month, and year above mentioned.

(*Signed.*)

Second Report—Analysis of the stains of blood and semen:—

We, the undersigned, A. C., chemist, etc., directed by an order of M., the judge of instruction, D., dated the ——, to the effect that, conjointly with M., the Doctor N., we should proceed to an analysis of the stains on the shift which was worn by the girl M. at the moment when, according to her declaration, violence on her person was committed, and determine the nature of these stains; an oath having been previously taken by us before this magistrate, we received from him a package containing the said shift, and we adjourned immediately to our laboratory, where, in concert with Doctor N., and in presence of Mr. M., the father of the young girl, who recognized the identity of the seals, we have ascertained—

That there were on the hinder part of the shift a great number of stains, occupying about 20 square centimetres, and seeming to be made with pure blood, or that slightly mixed with serosity; that several other smaller spots, elongated and of a brownish-yellow color, were evidently made by fecal matters.

That on the front of the shift, at its lower and middle part, there was a yellowish-gray stain, 7 or 8 centimetres in diameter, and some other smaller ones of the same appearance; that the linen had at these parts a greater consistence than common, and, as it were, starched; that the odor had in it nothing characteristic.

We then proceeded to the chemical operations necessary to determine the nature of these stains:—

1. Two of these stains, which seemed to be made by blood, were cut into several pieces, then introduced into distilled water in a close tube, which was so arranged as to be immersed in the fluid without touching the bottom or the sides of the tube. At the end of about two hours' maceration, reddish streaks, formed by the coloring matter, were deposited, and imparted a red color to the lower part of the fluid; the linen, which was discolored, exhibited only a very thin coat of soft grayish substance, somewhat elastic, soluble in potash, and this last in solution gave, by chlorine and a little chlorhydric acid, flocculi of coagulated matter, a union of phenomena which characterizes fibrin.

The liquid in which the coloring matter was deposited, on being filtered, (on a small filter previously moistened,) and then heated gradually by the flame of a spirits of wine lamp, became turbid and discolored, at the same time that there were formed reddish-gray flocculi. Two drops of a solution of potassa having been added, these flocculi disappeared, the liquid became clear and presented *a greenish tint seen by reflected light, and a reddish tint by refraction;* then, in passing a current of gaseous chlorine through the liquid and pouring on it afterwards some drops of chlorhydric acid, the albuminous flocculi were again formed; phenomena these which the coloring matter of the blood could alone produce.

2. The front part of the shift, the yellowish gray and pasty spots of which seemed to us to be caused by semen, was cut into small pieces. One of the stained pieces, subjected to great heat, assumed a more decidedly yellowish (yellow-fawn) tint. Two other pieces were placed in the upper part of a test tube containing distilled water, which was heated to the point of ebullition; the linen moistened by the vapor gave out a strong spermatic odor, blended with a weak smell of lye. Four other pieces were introduced into a small closed tube, 22 millimetres in diameter, holding distilled water; after three hours of maceration, the liquid, like the linen, exhaled a spermatic odor, and these pieces of linen were sticky. After squeezing out all the fluid they were dried by a moderate heat, and became again firm and slightly starch-like.

The fluid in which the maceration took place was cloudy, and contained small filaments, which we ascertained to come from the linen; although filtered several times, (the filter having been previously moistened,) it could not be obtained in a perfectly limpid state. The residual filtered liquid, put into a watch-crystal, and this into a sand-bath, and evaporated by a mild heat to the point of desiccation, gave out more and more a spermatic odor, and deposited a glutinous substance, which formed on the surface of the glass a shining and transparent lining. A little distilled water poured on this coat and agitated with a glass rod dissolved only a part of it. The insoluble portion, which was adherent to the end of the rod, on being heated by a solution of potash, was completely dissolved. The water charged with the soluble portion treated *by alcohol* was but slightly disturbed; another portion treated *by nitric acid* exhibited a barely sensible cloudiness. These two last tests, particularly, proved to us that the matter submitted to analysis was really semen, for alcohol precipitates abundantly the other liquids of the secretions, and nitric acid gives rise in them to flocculent precipitates, which for the most part collect at the bottom of the vessel.

Finally, in order to obtain a more complete proof of the presence of semen, we had to determine, by the aid of the microscope, the presence of the essential elements of the seminal fluid. We cut off from the stained part of the linen a strip of about a centimetre (nearly five lines) in width, the end of which we dipped in pure water contained in a watch-glass; the fluid soon rose, by virtue of capillary attraction, on the strip, and moistened the stain, which swelled up, and then gradually regained its thickness and the appearance which it had when in its original state.

At the end of half an hour we scraped very lightly with a scalpel the swelled stain, and the matter thus detached from it was placed on the object-glass of a microscope; we brought it to a state of somewhat greater division by agitating it in a portion of pure water, and then placed it under the microscope. We observed at first small whitish filaments coming from the tissue of the strips, and which were removed by the scraping; and then, in the middle of irregular corpuscles of multiform granules, we were able to see distinctly the spermatozoa; some of them entire, grayish and transparent, and of the length of about 5 to 6 centimetres of a millimetre, consisting of a large and somewhat flattened head and a long cylindrical appendix, narrower and becoming thinner from its point of origin; others broken up, but still easily recognized by the form and aspect of their head.

From the preceding facts and experiments, we conclude—

1. That the stains on the hinder part of the shift of the girl M. are made, some of them by fecal matter, and almost all of them by blood mixed with a little serosity.

2. That the stains observed on the front part of the shift are owing to semen.

We certify, etc.

These two practical lessons, in the form of "Reports," showing the manner of proceeding which the science now exacts of medical legists, form a suitable termination to the history and outlines of medical jurisprudence, such as we have endeavored to trace in these pages. We should have looked to an indefinitely future time for furnishing us with an opportunity to bring out, with some degree of prominence, certain cardinal questions yet untouched, did not immediate occasion offer itself, which demands an early resumption of the subject, as a matter of justice to the requirements of American literature. After we had made considerable progress in the present article, and shaped its chief features, we were favored with nearly a perfect copy, in sheets, of a new edition of Beck's Medical Jurisprudence, from the press of J. B. Lippincott & Co., which, in its entireness, will probably be issued as soon as the forthcoming number of our Journal. Of the general merits of this work we have spoken in a preceding page. It will be our duty, at an early day, to enlarge on these, and thus be enabled to handle a variety of topics of practical and abiding interest in a science which has not gained due attention from medical men, nor enlisted the services of the governors and teachers in our medical schools, notwithstanding its rich literature, its rapidly growing importance, and the frequent calls for a knowledge of its principles and an application of its details.

